# Ultrasound Tracking Reveals Progressive Regional Strain Differences in Human Achilles Tendons During Fatigue Loading

**DOI:** 10.64898/2026.09.09.750111

**Published:** 2026-09-15

**Authors:** Sittinon Nuethong, Josh R. Baxter

## Abstract

Ultrasound is commonly used to assess structural changes in symptomatic Achilles tendons, but quantitative biomechanical metrics for progressive tendon deterioration remain limited. The goal of this study was to develop and validate an automated ultrasound tracking algorithm for regional tendon deformation and evaluate strain progression in survived and ruptured tendons during fatigue loading. We hypothesized that maximum strain, average strain, and strain heterogeneity would exhibit different trajectories between groups. Ten cadaveric Achilles tendons underwent cyclic loading with stress tests every 500 cycles until rupture or 150,000 cycles. Ultrasound images acquired during stress tests were analyzed using an automated tracking algorithm to generate spatially resolved regional strain fields. Ultrasound-derived bulk strain was highly correlated with actuator-derived strain in survived (R ^2^ = 0.968 ± 0.017) and ruptured tendons (R ^2^ = 0.972 ± 0.014). Maximum and average longitudinal strains progressively diverged between groups across fatigue life (Group x FatigueLife: p = 0.003 and p < 0.0001, respectively). During the first 10,000 cycles, average strain decreased in survived tendons (β = −0.0268%, p = 0.0215) but not ruptured tendons (β = 0.0147%, p = 0.1197), with a significant Group x Cycle interaction (p = 0.0061). This study demonstrates that the algorithm quantified Achilles tendon deformation with high fidelity and enabled spatially resolved strain assessment throughout fatigue loading. Maximum and average strain followed different trajectories between groups, whereas strain heterogeneity did not. Early differences in tendon biomechanics suggest that regional strain behavior may change before pronounced differences in absolute magnitude develop.

## Full Text Availability

The license terms selected by the author(s) for this preprint version do not permit archiving in PMC. The full text is available from the preprint server.

